# Identification of Prognostic Molecular Features in the Reactive Stroma of Human Breast and Prostate Cancer

**DOI:** 10.1371/journal.pone.0018640

**Published:** 2011-05-18

**Authors:** Anne Planche, Marina Bacac, Paolo Provero, Carlo Fusco, Mauro Delorenzi, Jean-Christophe Stehle, Ivan Stamenkovic

**Affiliations:** 1 Institute of Pathology, CHUV, and Faculty of Biology and Medicine, University of Lausanne, Lausanne, Switzerland; 2 Department of Genetics, Biology and Biochemistry, University of Turin, Turin, Italy; 3 Swiss Institute of Bioinformatics, Lausanne, Switzerland; University of Birmingham, United Kingdom

## Abstract

Primary tumor growth induces host tissue responses that are believed to support and promote tumor progression. Identification of the molecular characteristics of the tumor microenvironment and elucidation of its crosstalk with tumor cells may therefore be crucial for improving our understanding of the processes implicated in cancer progression, identifying potential therapeutic targets, and uncovering stromal gene expression signatures that may predict clinical outcome. A key issue to resolve, therefore, is whether the stromal response to tumor growth is largely a generic phenomenon, irrespective of the tumor type or whether the response reflects tumor-specific properties. To address similarity or distinction of stromal gene expression changes during cancer progression, oligonucleotide-based Affymetrix microarray technology was used to compare the transcriptomes of laser-microdissected stromal cells derived from invasive human breast and prostate carcinoma. Invasive breast and prostate cancer-associated stroma was observed to display distinct transcriptomes, with a limited number of shared genes. Interestingly, both breast and prostate tumor-specific dysregulated stromal genes were observed to cluster breast and prostate cancer patients, respectively, into two distinct groups with statistically different clinical outcomes. By contrast, a gene signature that was common to the reactive stroma of both tumor types did not have survival predictive value. Univariate Cox analysis identified genes whose expression level was most strongly associated with patient survival. Taken together, these observations suggest that the tumor microenvironment displays distinct features according to the tumor type that provides survival-predictive value.

## Introduction

It is widely recognized that tumor progression and metastasis are intimately linked to tissue remodeling resulting from tumor cell interactions with the host tissue stroma. In normal epithelial tissues, the basement membrane provides a natural barrier between epithelial cells and the stroma. Proliferation of transformed epithelial cells is therefore initially confined to the epithelial compartment, leading to the development of a *carcinoma in situ*. Invasion is heralded by degradation of the tumor cell basement membrane, recently shown to be mediated primarily by membrane-bound matrix metalloproteinases (MT-MMPs) [1]. Subsequent to penetration of the basement membrane, tumor cells engage for the first time in physical contact with the extracellular matrix (ECM) and stromal cells, including fibroblasts, leukocytes, dendritic cells and endothelial cells, triggering cross-talk between tumor and stromal cells that has profound consequences on local tumor growth and tumor cell dissemination [2], [3], [4].

The sequence of events that occur following tumor cell irruption into the host tissue stroma is difficult to define because several events are likely to occur simultaneously. However, evidence suggests that cytokines, chemokines and proteolytic enzymes secreted by tumor cells participate in local macrophage, fibroblasts and endothelial cell activation and recruitment of a variety of leukocyte subsets [5], [6]. Activated macrophages and recruited leukocytes in turn secrete their own repertoire of cytokines, chemokines and proteolytic enzymes, leading to ECM degradation, which results in the release of a host of sequestered growth factors [7], [8], [9]. Some of these growth factors participate in promoting angiogenesis whereas others stimulate fibroblasts and myofibroblasts to synthesize and secrete ECM proteins [2], [5], [6]. The overall process is virtually indistinguishable from the remodeling that characterizes tissue repair following injury [10]. However, the released growth factors and ECM degradation products provide resources that ensure tumor cell survival, proliferation and migration, which in turn perpetuate tissue remodeling, leading to the notion that invasive tumors behave as “wounds that never heal” [11].

Tumor-associated stromal reactions vary both in amplitude and composition according, at least in part, to the tumor type. Most carcinomas display some degree of stromal reaction, which in some tumors, particularly breast, prostate and pancreatic carcinoma, can be associated with massive ECM deposition, referred to as desmoplasia. Because tissue remodeling provides a means for tumor cells to grow and disseminate, it is widely held that rational anticancer therapeutic design should target not only tumor cells but the reactive stroma as well [3], [4], [12]. It follows that understanding tumor-stroma cross-talk at the molecular level and identification of molecular events whose disruption may destabilize tumor growth will constitute key steps toward therapeutic control of cancer growth. Several approaches have been used to address the stromal response to invasive cancer growth, including gene expression profile analysis of microdissected reactive stroma in human [13], [14], [15] and murine [16] tumors; gene expression analysis in defined FACS-sorted breast cancer stromal cell subsets [17]; development of new bioinformatics methods that decompose the gene expression signal originating from the entire tumor into multiple independent signatures allowing identification of those emanating from the stroma [18]; and modeling inducible tumor development to study tumor-host interactions as a function of time during tumor progression. Together, these studies have identified several candidate stroma-derived molecules that compose gene expression signatures relevant to cancer progression and metastases [3], [13], [14], [15], [16], [18], [19]. However, all of these studies have focused on the stromal reaction of a particular tumor type, and although the identified reactive stromal gene expression signatures are reported to bear prognostic significance to the tumors they are associated with, it is unclear whether different tumor types share reactive stromal gene expression signatures or whether they elicit distinct responses.

In the present work we focused on the analysis of gene expression signatures of human breast and prostate cancer stroma in an effort to determine the degree of similarity among stromal reactions to different invasive cancer types and identify candidate deregulated genes common to tumor invasion irrespective of tumor origin. Our results reveal distinct stromal gene expression signatures in human breast and prostate cancer, each of which is predictive of poor prognosis of its respective tumor type, and identify a small deregulated gene set common to both tumor types that, by contrast, is not predictive of patient survival.

## Results

### Patient sample selection

Breast and prostate cancer patients were selected according to the following criteria: availability of both tumor and normal tissue for each patient; presence of an adequate amount of stroma in both normal and tumor tissues for efficient microdissection; absence of chemotherapy and/or radiotherapy and presence of a comparable inflammatory reaction, as assessed by histological analysis, to limit variability among samples. To ensure reliable statistical analysis at least six patients per cancer type with defined histopathological characteristics were included (Table S1). All breast cancer patients had primary tumors with an invasive component that was at least 0.5 cm in the greatest dimension and five out of six patients presented lymph node metastasis and were estrogen receptor (ER)-positive (90–100%). All prostate cancer patients presented primary invasive tumors involving both lobes of the prostate, with a Gleason score≥7 and no lymph node metastasis (pN0), thus constituting a homogeneous group. Both normal and tumor tissues were hematoxylin-eosin (H&E)-stained to assess tissue morphology prior to microdissection.

The selected candidate samples were subsequently stained using an anti-multi-cytokeratin antibody to identify tumor cells within tissue sections (Figure S1, panels A and B) and an anti-vimentin antibody to identify the stromal compartment (Figure S1 panel C). Extensive stromal areas within tumor tissue sections were found to be free of invading tumor cells and were thus amenable to microdissection. Normal and tumor tissue sections of the breast and prostate patients were subjected to laser capture microdissection (LCM) for selective analysis of the stromal compartment (Figure S2). Generally, 20 to 100 ng of total RNA were extracted from microdissected samples and subjected to mRNA amplification prior to hybridization to Affymetrix microarrays.

### Breast and prostate cancer display distinct stromal responses

**Figure 1:**
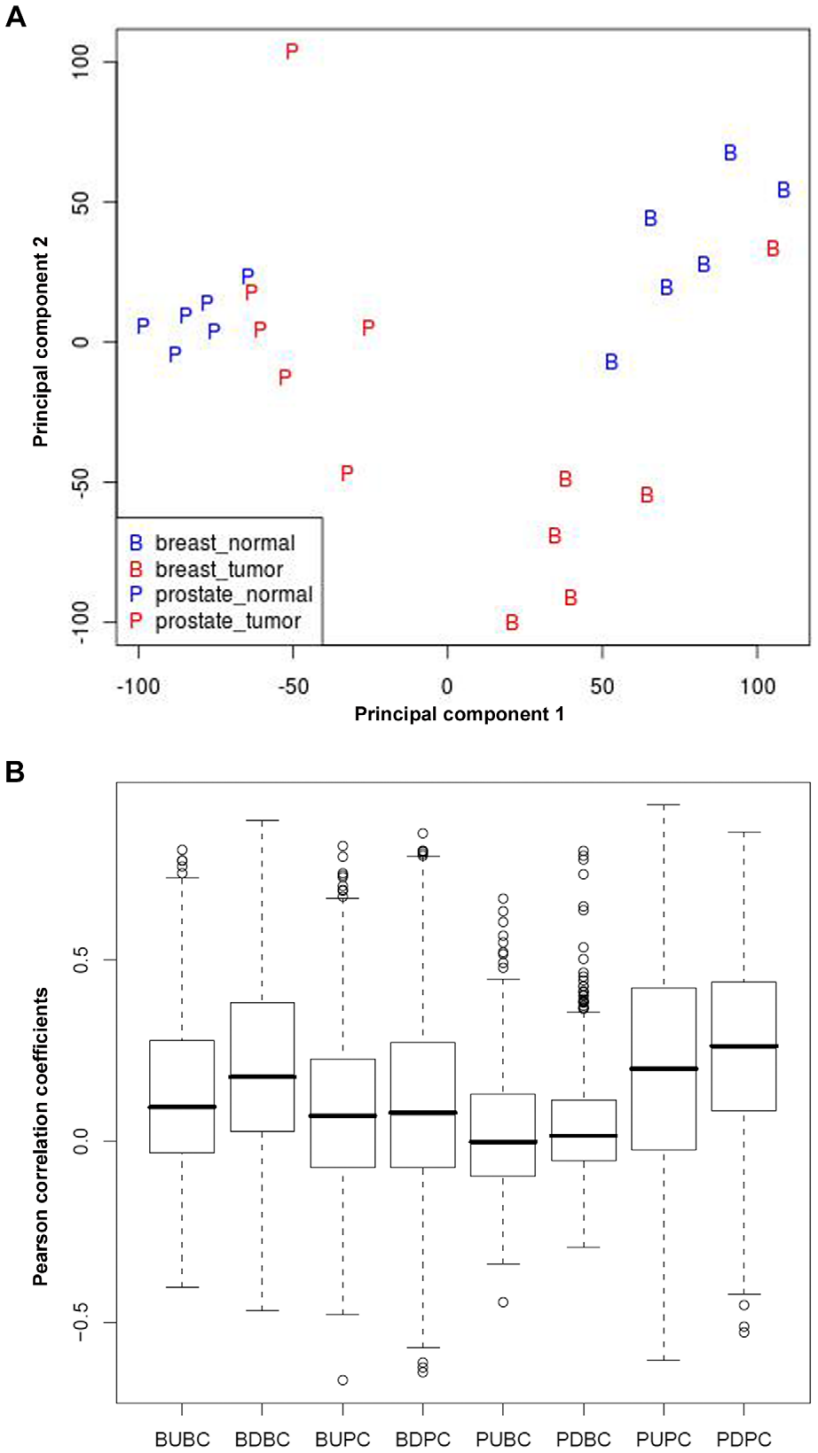
Tumor-specific stromal responses displayed by breast and prostate cancer. A, PCA shows that breast and prostate tumors have a distinct stromal reaction to tumor invasion that can be used to classify cancer patients. B, pairwise correlation analysis showing a higher correlation of breast stromal genes with breast data than with prostate data and vice versa.

The global gene expression profile of microdissected stroma obtained from breast and prostate specimens was first analyzed using Principal Component Analysis (PCA). The projection of the stromal expression profiles on the two first components is shown in Figure 1A. Notwithstanding some outliers, the figure demonstrates a clear distinction in stromal expression profiles between breast and prostate tumors and also between tumor and normal samples of each tissue type. The figure also suggests that the overall stromal response in breast cancer is stronger than in prostate cancer. We concluded that breast and prostate tumors have a distinct stromal reaction to tumor invasion that may be successfully used for classifying cancer patients. In addition, we defined genes sets labeled BU, BD, PU and PD containing the genes represented by probesets that are up or downregulated in breast and prostate tumor stroma compared to the corresponding normal stroma at FDR 5% and 10% cutoffs, respectively, and at least a 2-fold change in expression level. We used different FDR cutoffs for breast and stroma to obtain lists of differentially expressed genes of comparable size. The fact that we had to use a higher FDR in the case of prostate cancer confirms that the overall stromal response is weaker than in breast cancer. Pearson correlation between any pair of different genes in each of these stromal gene sets calculated in the ExpO consortium breast and prostate subsets shows a better correlation of the breast stromal genes with breast data (BU: 0.09/BD: 0.18) than with prostate data (BU: 0.07/BD: 0.08). Similarly, prostate stromal genes show better correlation with prostate data (PU: 0.20/PD: 0.26) than with breast data (PU: 0.00/PD: 0.01) (Figure 1B).

### Differentially expressed genes between tumor and normal stroma

The genes sets BU, BD, PU and PD defined above contained 181 and 462 statistically relevant probes for BU and BD, respectively, (FDR 5%, Table S2), and 154 and 165 for PU and PD, respectively, (FDR 10%, Table S3). Fourteen randomly chosen genes within the lists were validated by quantitative real-time PCR (Figure S3).

### Genes specific to the stromal reaction of breast tumors

A selection of genes found to be differentially expressed between tumor and normal stroma of breast cancer patients are listed in Table 1. Stromal reaction to invasive breast carcinoma was associated with increased expression of genes encoding ECM components, proteolytic enzymes and adhesion receptors, including *COL11A1*, *COL10A1*, *COMP*, *MMP11*, *FN1* and *MFAP2*, consistent with the abundant stromal remodeling observed by histology. Genes encoding components of the ECM, including *TNXB* and *MATN2* were identified among downregulated transcripts, together with other participants in tumor progression, including growth factors, such as *FIGF* and growth factor receptors, such as *TGFBR3*.

**Table 1:** Selection of differentially expressed genes between tumor and normal breast stroma (FDR<0.05, |M|≥2).

Gene symbol	Gene description	logFC	Adjusted P-value
***Upregulated genes in tumor stroma***			
COL11A1	Collagen, type XI, alpha 1	7.3	6.0E-03
COL10A1	Collagen, type X, alpha 1(Schmid metaphyseal chondrodysplasia)	6.0	1.2E-02
COMP	Cartilage oligomeric matrix protein	4.9	1.6E-02
INHBA	Inhibin, beta A	4.8	8.0E-03
CXCL10	chemokine (C-X-C motif) ligand 10	3.9	4.8E-02
SULF1	Sulfatase 1	3.7	2.4E-02
SDC1	Syndecan 1	3.4	2.4E-02
MMP11	Matrix metallopeptidase 11 (stromelysin 3)	3.2	2.6E-02
F2RL1	Coagulation factor II (thrombin) receptor-like 1	3.1	3.1E-02
CDKN2B	Cyclin-dependent kinase inhibitor 2B (p15, inhibits CDK4)	3.1	2.3E-02
RUNX2	Runt-related transcription factor 2	2.7	4.7E-02
CDKN2A	Cyclin-dependent kinase inhibitor 2A (melanoma, p16, inhibits CDK4)	2.6	3.1E-02
CADM1	Cell adhesion molecule 1	2.5	6.0E-03
P4HA3	Procollagen-proline, 2-oxoglutarate 4-dioxygenase (proline 4-hydroxylase), alpha polypeptide III	2.4	3.8E-02
FN1	Fibronectin 1	2.4	3.8E-02
NRG1	Neuregulin 1	2.2	4.7E-02
MFAP2	Microfibrillar-associated protein 2	2.2	4.9E-02
RUNX1	runt-related transcription factor 1 (acute myeloid leukemia 1; aml1 oncogene)	2.1	1.6E-02
***Downregulated genes in tumor stroma***			
CD36	CD36 molecule (thrombospondin receptor)	−4.9	3.2E-03
FIGF	C-fos induced growth factor (vascular endothelial growth factor D)	−4.8	1.0E-02
KLF4	Kruppel-like factor 4 (gut)	−3.9	2.4E-02
MATN2	Matrilin 2	−3.7	2.5E-02
LIFR	Leukemia inhibitory factor receptor alpha	−3.5	1.2E-02
EMCN	Endomucin	−3.3	2.7E-02
GPC3	Glypican 3	−3.2	1.1E-02
FOSB	FBJ murine osteosarcoma viral oncogene homolog B	−3.2	1.9E-02
IL33	Interleukin 33	−3.1	4.9E-02
MEG3	Maternally expressed 3	−3.1	7.4E-03
TGFBR3	Transforming growth factor, beta receptor III	−3.1	6.1E-03
RHOJ	Ras homolog gene family, member J	−3.1	2.6E-02
DLC1	Deleted in liver cancer 1	−3.0	3.1E-02
TNXB	Tenascin XB	−2.9	5.0E-03
ANK2	Ankyrin 2, neuronal	−2.8	4.3E-02
NOVA1	neuro-oncological ventral antigen 1	−2.6	1.6E-02
ENPP2	Ectonucleotide pyrophosphatase/phosphodiesterase 2 (autotaxin)	−2.6	3.8E-02
LEPR	Leptin receptor	−2.6	6.0E-03

### Genes specific to the stromal reaction of prostate cancer

A distinct selection of genes found to be differentially expressed in the tumor stroma of prostate cancer patients compared to their normal tissue counterparts is shown in Table 2. In contrast to breast tumor stroma, the stromal reaction to invasive prostate cancer displayed fewer genes involved in tissue remodeling but a higher number of genes belonging to defined signaling pathways, including members of the Wnt signaling pathway (*SFRP1*, *RSPO3*). Several transcription factors, including *NKX3-1*, *HOXB13*, *HOXC6*, *HOXD11* and *HOXD13*, were also found to have deregulated expression in the stromal reaction to invasive prostate tumors.

**Table 2:** Selection of differentially expressed genes between tumor and normal prostate stroma (FDR<0.10, |M|≥2).

Gene symbol	Gene description	logFC	Adjusted P-value
***Upregulated genes in tumor stroma***			
PRAC	Prostate cancer susceptibility candidate	4	1.4E-02
ASPN	Asporin	3.8	2.5E-02
CTHRC1	Collagen triple helix repeat containing 1	3.7	7.5E-02
TARP	TCR gamma alternate reading frame protein	3.4	1.1E-02
AGR2	Anterior gradient homolog 2 (Xenopus laevis)	3.2	5.3E-02
POSTN	Periostin, osteoblast specific factor	3.2	9.8E-02
ESR1	estrogen receptor 1	3.2	6.6E-02
NKX3-1	NK3 homeobox 1	3.2	4.1E-02
HOXB13	Homeobox B13	2.8	4.6E-02
SFRP1	Secreted frizzled-related protein 1	2.8	6.3E-02
BMPR1B	Bone morphogenetic protein receptor, type IB	2.7	4.8E-02
FOLH1	Folate hydrolase (prostate-specific membrane antigen) 1	2.7	9.9E-02
RSPO3	R-spondin 3 homolog (Xenopus laevis)	2.3	5.7E-02
PKP2	Plakophilin 2	2.3	6.9E-02
ERG	V-ets erythroblastosis virus E26 oncogene homolog (avian)	2.3	5.3E-02
TSPAN1	Tetraspanin 1	2.2	3.2E-02
HOXC6	Homeobox C6	2	3.0E-02
GREB1	GREB1 protein	2.0	6.9E-02
***Downregulated genes in tumor stroma***			
NELL2	NEL-like 2 (chicken)	−4.6	6.2E-02
BMP5	Bone morphogenetic protein 5	−4.5	2.9E-02
PENK	Proenkephalin	−4.2	5.4E-02
GPM6A	Glycoprotein M6A	−4.1	1.2E-02
DKK1	Dickkopf homolog 1 (Xenopus laevis)	−3.4	9.8E-02
PTGS1	Prostaglandin-endoperoxide synthase 1 (prostaglandin G/H synthase and cyclooxygenase)	−3.1	9.4E-02
SEMA3E	Sema domain, immunoglobulin domain (Ig), short basic domain, secreted, (semaphorin) 3^E^	−2.8	3.0E-02
FOXQ1	Forkhead box Q1	−2.8	5.3E-02
DPT	Dermatopontin	−2.7	9.4E-02
ARHGAP28	Rho GTPase activating protein 28	−2.7	8.8E-02
HOXD13	homeobox D13	−2.7	6.6E-02
TSLP	Thymic stromal lymphopoietin	−2.4	2.4E-02
PRKCB1	Protein kinase C, beta 1	−2.4	2.9E-02
PTGDS	Prostaglandin D2 synthase 21 kDa (brain)	−2.3	9.8E-02
HAPLN1	Hyaluronan and proteoglycan link protein 1	−2.3	8.6E-02
GPR133	G protein-coupled receptor 133	−2.1	8.0E-02
PGF	Placental growth factor	−2.0	8.8E-02
HOXD11	Homeobox D11	−2.0	8.8E-02

### Genes common to the stromal reaction of both tumor types

Although PCA showed a clear separation of breast and prostate patients, suggesting a limited overlap between the lists of breast and prostate stromal genes, we nevertheless attempted to compare the two lists in order to identify genes that might be common to the stromal reaction of both tumor types. Using an FDR of 15% for both breast and prostate analyses, we identified 20 upregulated (P = 1.3E-03, Fisher's exact test) and 28 downregulated (P = 2.4E-05) common genes (Table 3). Several of the upregulated genes encoded adhesion receptors, secreted proteins and cytoskeletal components, including *CDH11*, *POSTN* and *MYO5B*, along with *RUNX1*, a master regulator of differentiation processes in different tissues implicated in cell transformation and tumor progression [20], [21]. Several of the downregulated genes encoded enzymes implicated in metabolic processes including *BCO2*, *GLT25D2*, *GSTM5*, *ASPA* and *PTGDS*. Interestingly, the hepatic leukemia factor (*HLF*), a member of bZIP transcription factor family known to regulate the expression of *RUNX1*, was also found to be downregulated.

**Table 3:** Genes common to the stromal reaction of breast and prostate cancer patients (FDR 15%).

Gene symbol	Gene description
***Upregulated genes in the tumor stroma***	
ABCC4	ATP-binding cassette, sub-family C (CFTR/MRP), member 4
C11orf75	chromosome 11 open reading frame 75
CDH11	cadherin 11, type 2, OB-cadherin (osteoblast)
ENC1	ectodermal-neural cortex (with BTB-like domain)
ESRP2	epithelial splicing regulatory protein 2
GOLM1	golgi membrane protein 1
KIAA0101	KIAA0101
MYO5B	myosin VB
NDUFS8	NADH dehydrogenase (ubiquinone) Fe-S protein 8, 23 kDa (NADH-coenzyme Q reductase)
NNMT	nicotinamide N-methyltransferase
NTM	neurotrimin
PBRM1	polybromo 1
PDLIM5	PDZ and LIM domain 5
POSTN	periostin, osteoblast specific factor
RUNX1	runt-related transcription factor 1
SERP1	stress-associated endoplasmic reticulum protein 1
SORD	sorbitol dehydrogenase
SPATS2L	spermatogenesis associated, serine-rich 2-like
VOPP1	vesicular, overexpressed in cancer, prosurvival protein 1
YARS	tyrosyl-tRNA synthetase
***Downregulated genes in the tumor stroma***	
ADAMTS5	ADAM metallopeptidase with thrombospondin type 1 motif, 5
ADCYAP1R1	adenylate cyclase activating polypeptide 1 (pituitary) receptor type I
ANKDD1A	ankyrin repeat and death domain containing 1A
ASPA	aspartoacylase (Canavan disease)
BCO2	beta-carotene oxygenase 2
C16orf89	chromosome 16 open reading frame 89
CFD	complement factor D (adipsin)
CLEC3B	C-type lectin domain family 3, member B
ETS2	v-ets erythroblastosis virus E26 oncogene homolog 2 (avian)
GARNL3	GTPase activating Rap/RanGAP domain-like 3
GLT25D2	glycosyltransferase 25 domain containing 2
GPM6A	glycoprotein M6A
GPR133	G protein-coupled receptor 133
GSTM5	glutathione S-transferase mu 5
HLF	hepatic leukemia factor
ITM2A	integral membrane protein 2A
KIAA1377	KIAA1377
NAP1L5	nucleosome assembly protein 1-like 5
PENK	proenkephalin
PHACTR2	phosphatase and actin regulator 2
PPARG	peroxisome proliferator-activated receptor gamma
PPL	periplakin
PTGDS	prostaglandin D2 synthase 21kDa (brain)
PTGFR	prostaglandin F receptor (FP)
THSD7A	thrombospondin, type I, domain containing 7A
TJP2	tight junction protein 2 (zona occludens 2)
TRERF1	transcriptional regulating factor 1
ZNF10	zinc finger protein 10

Common upregulated genes: P = 0.0013, common downregulated genes: P = 2.4E-05.

Comparison to datasets from studies on human breast and pancreatic and murine prostate cancer revealed a high degree of similarity between upregulated genes in our breast cancer patient stroma and upregulated genes in the Ma et al. [13] and Bauer et al. study of breast tumors [22] as well as in the Binkley et al. study of the stromal response to pancreatic cancer [15] (Table 4). Significant similarity was also found with the mouse stromal response to neuroendocrine prostate cancer growth [16]. The prostate cancer stromal signature was also significantly related to these four datasets, albeit to a lesser degree than the breast cancer signature (Table 4). As expected, our breast cancer stromal signature was more closely related to the two breast signatures than our prostate cancer stromal signature. In addition, both our breast and prostate cancer stromal signatures displayed similarity with pancreatic cancer and mouse neuroendocrine prostate cancer stroma signatures. Closer examination of the signatures, however, revealed that the similarity resided primarily among genes implicated in tissue remodeling.

**Table 4:** Comparison of upregulated breast and prostate genes identified in the present study with published stromal signatures.

	Stroma-related gene expression studies			
Present study (FDR 15%)	Ma *et al.* (breast carcinoma)	Bauer *et al.* (breast carcinoma)	Binkley *et al.* (pancreatic carcinoma)	Bacac *et al.* (prostate carcinoma, mouse)
**Breast stromal genes**	8.1E-22	2.4E-04	9.8E-16	1.3E-07
**Prostate stromal genes**	0.086	0.02	3.8E-03	8.3E-03

**Figure 2:**
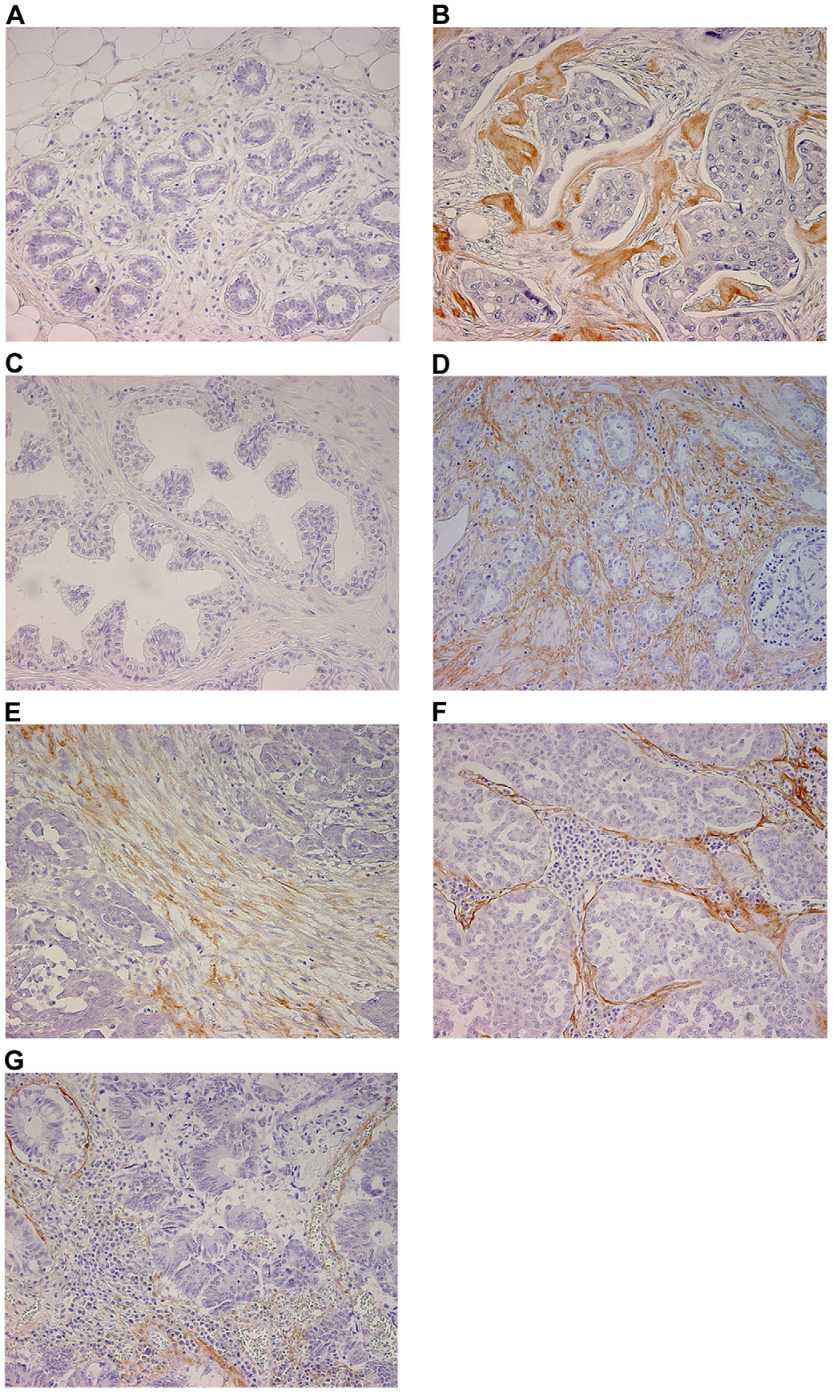
Representative images of periostin expression in normal and tumor tissues. A, normal breast tissue. B, breast carcinoma. C, normal prostate tissue. D, prostate carcinoma. E, ovarian carcinoma. F, lung carcinoma. G, colon carcinoma. Magnification: 200×.

Periostin (POSTN), found to be upregulated in both breast and prostate cancer stroma, was selected for immunohistochemical validation in a panel of human tumors known to be associated with a prominent stromal reaction (breast, prostate, ovary, colon and lung carcinoma). Representative images shown in Figure 2 confirm the increase of POSTN expression in the stromal compartment of breast and prostate tumor samples (panels B and D, respectively), compared to their normal counterparts (panels A and C, respectively). Intense POSTN expression was also observed in the stroma of ovarian carcinoma (panel E), as well as in lung and colon carcinoma where it was concentrated at the interface between the tumor epithelial cells and the stromal compartment that presented a robust inflammatory reaction (panels F and G, respectively). It is noteworthy that POSTN was not expressed in the tumor cells of the samples analyzed.

### Prognostic value of specific and common stromal signatures

**Figure 3:**
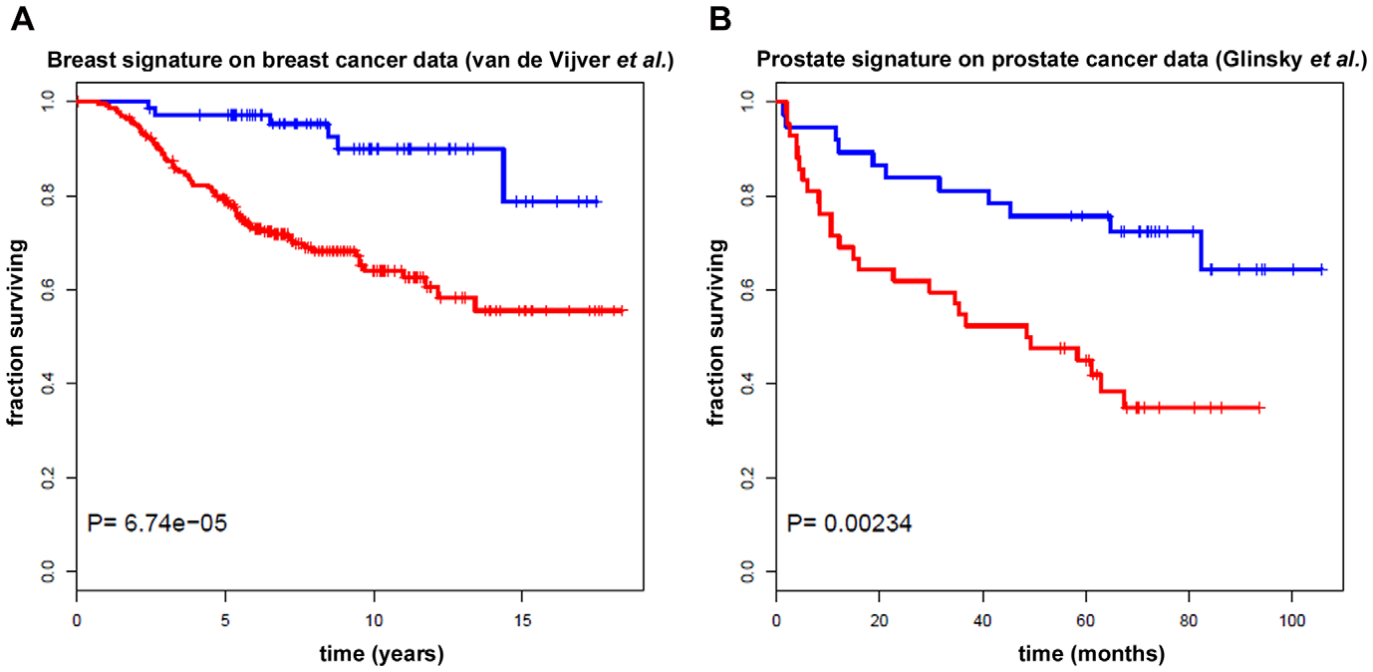
Kaplan-Meier survival analysis. A, Kaplan-Meier survival analysis of early-breast carcinoma patients (van de Vijver et al.) and B, prostate carcinoma patients (Glinsky et al.) obtained using breast and prostate stromal genes respectively (FDR 15%), showing that the two groups of patients differ significantly in their overall survival. Red, poor prognosis group; blue, good prognosis group.

Genes identified in breast and prostate stromal reactions (FDR 15%) were assessed for their survival-predictive ability using publicly available datasets of human cancer patients. For each dataset, Pearson correlation-based hierarchical clustering was first used to divide patients into two groups based only on the expression profiles of breast and prostate stromal genes. Kaplan-Meier analysis and log-rank test were then used to determine whether the two groups of patients thus defined showed statistically significant differences in terms of survival. Figure 3A represents the results of Kaplan-Meier survival analysis obtained using breast stromal genes (FDR 15%) on 295 early-stage breast carcinoma patients [23]. The two groups of patients, obtained after hierarchical cluster analysis using stromal genes, differed significantly in their overall survival (P = 6.74e-05), indicating that the breast stromal genes had survival-predictive value for breast cancer patients.

Similarly to breast stroma, prostate stromal genes also displayed statistically significant survival-predictive ability (P = 0.002) on 79 prostate carcinoma patients [24], (Figure 3B) if only genes with base 2 logarithmic fold change |M|>2 are included in the signature. By contrast, genes common to breast and prostate cancer stroma did not display statistically significant prognostic value for breast (23) (P = 0.773) or prostate (24) (P = 0.106) cancer.

### Univariate Cox analysis: identification of genes whose expression correlates most strongly with patient survival

Kaplan-Meier survival analysis indicated that the overall lists of breast and prostate cancer stromal genes had high prognostic value in human breast and prostate cancer datasets, respectively, but did not allow the identification of genes whose expression level is most strongly associated with patient survival. To address this issue, univariate Cox analysis was performed to correlate the level of gene expression with patient survival. For each gene, a z value was obtained, indicating the strength of the correlation between the level of gene expression and patient survival. Positive z values indicated that the expression level of a gene was associated with poor prognosis, while negative z values indicated correlation with good prognosis. A selection of genes associated with poor and good prognosis for breast ([23], Table 5) and prostate ([24], Table 5) cancer are shown. It is noteworthy that although the gene expression signature that was common to the stromal reaction of both breast and prostate carcinoma did not have any survival-predictive value, two individual genes within the common signature, *POSTN* and *RUNX1*, were associated with survival of patients with both tumor types. Interestingly, whereas periostin was associated with good survival in breast cancer patients, its overexpression was associated with poor prognosis in prostate cancer patients (Table 5).

**Table 5:** Cox analysis.

Gene symbol	Gene description	Z value
***Breast stromal genes***		
YARS	Tyrosyl-tRNA synthetase	4.4
ADAM19	ADAM metallopeptidase domain 19 (meltrin beta)	3.6
BMP2	Bone morphogenetic protein 2	3.5
SPP1	Secreted phosphoprotein 1	3.3
TNXB	Tenascin XB	2.5
EGFR	Epidermal growth factor receptor (erythroblastic leukemia viral (v-erb-b) oncogene homolog, avian)	2.4
NOVA1	Neuro-oncological ventral antigen 1	−3.2
XIST	X (inactive)-specific transcript (non-protein coding)	−2.4
INHBA	Inhibin, beta A	−2.4
POSTN	Periostin, osteoblast specific factor	−2.2
TGFBR3	Transforming growth factor, beta receptor III	−2.2
RUNX1	Runt-related transcription factor 1	−2.0
***Prostate stromal genes***		
HOXC6	Homeobox C6	3.9
SERP1	Stress-associated endoplasmic reticulum protein 1	3.3
CDH11	Cadherin 11, type 2, OB-cadherin (osteoblast)	2.5
BMPR1B	Bone morphogenetic protein receptor, typeIB	2.4
POSTN	Periostin, osteoblast specific factor	2.2
GREM1	Gremlin 1, cysteine knot superfamily, homolog (Xenopus laevis)	2.1
HOXD13	Homeobox D13	−3.8
GRIA1	Glutamate receptor, ionotropic, AMPA 1	−3.5
RUNX1	Runt-related transcription factor 1	−3.4
PTGDS	Prostaglandin D2 synthase 21 kDa (brain)	−3.0
GARNL3	GTPase activating Rap/RanGAP domain-like 3	−2.2
ESR1	Estrogen receptor 1	−2.0

Selection of breast and prostate stromal genes strongly associated with breast cancer patient survival (van de Vijver *et al.*) and prostate cancer patient survival (Glinsky *et al.*), respectively. Positive Z values indicate that the expression level of the gene is associated with poor prognosis, while negative Z values indicate correlation with good prognosis.

## Discussion

Breast and prostate cancer are the most common invasive cancers in women and men, respectively. Although these tumors arise in organs that are widely divergent in terms of anatomic localization, structure and physiological function, both organs require gonadal hormones for normal development. Accordingly, the corresponding tumors are hormone-dependent and display remarkable biological similarity. Based on this notion and the observation that both tumor types are usually accompanied by robust tissue remodeling, it is of interest to determine whether the elicited stromal response displays similar or distinct hallmarks. PCA performed using gene expression profiles of the analyzed samples revealed that the two tumor types had a distinct stromal reaction (Figure 1A). Breast cancer stroma was associated with genes encoding matrix components, including *COL11A1*, *COL10A1*, *COMP*, *MMP11*, *FN1*, *MFAP2*, *TNXB* and *MATN2*, consistent with the robust ECM remodeling frequently observed within breast tumors, whereas prostate cancer stroma was associated with deregulated expression of homeobox genes including *NKX3-1*, *HOXB13 HOXC6*, *HOXD11* and *HOXD13*, implicated in differentiation processes during development. Enhanced expression of these genes raises the interesting possibility that reactivation of developmental programs by prostate tumor stromal cells may contribute to the establishment of a more permissive microenvironment for tumor growth and progression.

Interestingly, a small subset of genes was found to be common to the stromal reaction of both tumor types and included, among others, genes encoding adhesion and cytoskeletal proteins (*CDH11*, *MYO5B*), a master regulator of differentiation processes, cell transformation, and tumor progression (*RUNX1*), as well as the osteoblast-specific factor periostin (*POSTN*). Several of the up and downregulated genes identified by microarray analysis were validated using *q*Real-time RT-PCR. Further validation of the relevance of the stromal genes was obtained from survival analysis using publicly available breast and prostate cancer patient datasets. Kaplan-Meier survival analysis revealed that the stromal genes identified in the present study clustered the cancer patients into two groups that differed significantly in their overall survival, underscoring their survival-predictive ability. It is noteworthy that the gene expression signature common to the stromal reaction of breast and prostate tumors did not carry prognostic value, suggesting that the “common” remodeling observed in several tumor types is not a key element in survival. Rather, tumor-specificity of the stromal reaction appears to be implicated in predicting evolution and survival.

Univariate Cox analysis further highlighted genes whose expression was most strongly associated with patient survival including, *POSTN* and *RUNX1* that were found to be common to the stromal reaction of both tumor types. Periostin was originally isolated as an osteoblast specific factor, and most of its physiologic functions take place at the epithelial-mesenchymal interface [25]. It is highly homologous to human β Ig-H3, a transforming growth factor β (TGF-β)-induced protein that promotes adhesion and spreading of fibroblasts [26]. Binding of periostin to α_V_β_3_, α_V_β_5_ or α_6_β_4_ integrins has been reported to promote invasion of tumor cells by enhancing cell survival via the Akt/PKB pathway [27], [28], [29]. POSTN was found to be overexpressed in several human cancers including ovarian [28], [30], colon [29], pancreatic [25], [27], breast [31], [32], lung cancer [33], and melanoma [34], with contradictory data concerning the identity of periostin-expressing cells (i.e. stroma, tumor cells or both).

In the present study, periostin was found to be upregulated and specifically localized to the breast and prostate tumor stroma compared to the normal stroma by immunohistochemistry. The presence of the periostin protein was also shown in the stroma of ovarian, colon and lung carcinoma.

The correlation between periostin expression and poor prostate cancer patient outcome is consistent with previous studies that identified periostin overexpression in several invasive tumor types [25], [28], [29], [34]. Recently, periostin was found to promote invasiveness of esophageal carcinoma [35]. However, another study reported a downregulation of POSTN in lung cancer tissues indicating a potential context-dependent tumor suppressor activity of POSTN [33] that could be in line with the association of POSTN overexpression with good prognosis in breast cancer patients observed in the present study.

Although the notion that tissue remodeling associated with tumor invasion facilitates subsequent tumor progression is widely accepted, the precise molecular features of the remodeling require elucidation if the stromal reaction is to be targeted by therapeutic means. It is therefore important to determine whether tumor invasion in and of itself induces a standard stromal reaction that varies only in amplitude among tumors or whether different tumor types induce distinct stromal reactions whose features are likely to have a bearing on the choice of therapeutic arsenal. The present study reveals that the stromal reaction to invasion by two unrelated tumor types bears distinctive features that are relevant to the prognosis of the respective tumors. By contrast, the gene signature found to be common to breast and prostate stromal reactions failed to show survival-predictive value. However, when Cox analysis was performed, two genes within the common signature, *RUNX1* and *POSTN*, were found to be associated with patient survival, providing potential therapeutic targets of interest. Periostin in particular seems to offer attractive therapeutic possibilities, as it is secreted and expressed selectively in tumor but not in normal stroma. Our study proposes periostin to be a novel stromal candidate marker of tumor prognosis that may also constitute potential therapeutic target in a broad range of carcinomas.

## Materials and Methods

### Patients and sample collection

Fresh frozen samples from six invasive breast and six invasive prostate tumors were obtained from the Institute of Pathology tissue bank, University Hospital Lausanne (CHUV) in compliance with institutional ethical regulations. Informed written consent was obtained from all patients involved in the study and approval was obtained from the ethics committee of the CHUV and Faculty of Biology and Medicine of the University of Lausanne.

### Laser capture microdissection

LCM slides were prepared from serial 6-µm-thick frozen tissue sections mounted on a polyvinyl nuclease free membrane (Molecular Machine&Industries, Glattbrugg, CH).

Tissue sections were fixed in ethanol 70% (30 sec), stained with Mayer's hematoxylin (10 sec) and eosin (30 sec), dehydrated in graded ethanol, treated with xylene and air-dried in a sterile laminar flow hood. Slides were microdissected immediately following staining using a μCut Laser Microdissector system (Nikon Eclipse TE200).

All steps and solutions were performed under RNase free conditions. All samples were subjected to histological examination in order to identify stromal regions free of tumor cells prior to microdissection.

### RNA extraction, amplification and microarray

Total RNA was extracted immediately following microdissection using the PicoPure RNA isolation kit (Arcturus, Mountain View, CA, USA) according to the manufacturer's instructions. RNA was quantified using a NanoDrop spectrophotometer (NanoDrop Technologies, Wilmington, Delaware, USA), and the concentration ranged between 20–100 ng/sample.

RNA quality was assessed using an Agilent RNA 6000 Pico Kit (Agilent Technologies, Germany). Only high quality RNA was subjected to two rounds of linear amplification using the MessageAmp II aRNA Amplification Kit (Ambion, USA) according to the manufacturer's instructions and amplified RNA (aRNA) was quantified using the RNA 6000 Pico Assay Kit. During the second round of amplification, biotin-labeled nucleotides were incorporated to obtain biotin-labeled aRNA required for Affymetrix microarray hybridization. GeneChip Human Genome U133 plus 2.0 Arrays (Affymetrix, UK) representing 47,000 different RNAs were used and the following steps performed by the DNA Array Facility of Lausanne (DAFL, http://www.unil.ch/dafl): fragmentation of aRNA, hybridization on the arrays, washing and scanning of the microarrays. The outputs of the scanning were CEL files containing a value representing the level of expression for each probesets from which expression measures in log2 were computed before subsequent statistical analysis.

### Statistical analysis

The RMA (Robust Multichip Average) algorithm was first applied to the microarray raw data to obtain gene expression data. All statistical analyses were performed using R and the Bioconductor suite (http://www.r-project.org/).

PCA was performed using the *prcomp* R function with default parameters.

Hierarchical cluster analysis was based on Pearson correlation between the samples. Differentially expressed genes between tumor and normal samples were identified with the *limma* package of Bioconductor, which applies empirical-based methods to a moderated t-statistic and takes multiple testing into account by providing an estimate of the false discovery rate (FDR). This analysis was performed in a paired way, .i.e. comparing tumor and normal samples from the same patient.

For the pairwise correlation analysis, the Pearson correlation was calculated in the ExpO breast and prostate subsets. Gene expression and annotation data from the ExpO consortium (http://www.intgen.org/expo/) were downloaded from GEO (GSE2109) in December 2008, including batches 1–16. The breast and prostate cancer subsets (354, respectively 83 samples) were extracted and processed separately with the RMA procedure (quantile normalization at probe-level data).

For comparison with published stromal signatures, multiple testing correction was done with the Bonferroni procedure. We re-analyzed the expression data of Ma *et al.* [13] to obtain a list of differentially expressed genes comparing invasive breast ductal carcinoma stroma versus normal stroma. For that we used the expression data deposited in GEO (series GSE14548) and performed a paired analysis of differential expression using *limma*. The probesets with FDR<1% were then selected and used for the comparison. We compared our upregulated stromal genes with the ones found upregulated in breast carcinoma-associated fibroblasts compared to normal mammary fibroblasts in Bauer *et al.* [22] We compared our data with the pancreatic cancer stroma genes set identified in Binkley *et al.* [15] For the comparison with the mouse study from Bacac *et al.* [16] we considered the list containing the mouse genes found to be upregulated in invasive compared to pre-invasive prostate tumor stroma. These genes were converted into human genes using HomoloGene (build 62) and taking into account only the mouse genes with a unique homologene human ortholog.

### Survival analysis of publicly available data

Publicly available gene expression data together with corresponding survival data for breast cancer and prostate cancer were obtained on-line. The breast data were directly downloaded from http://www.rii.com/publications/2002/nejm.html whereas the prostate data were provided by the authors as raw CEL files and normalized with the RMA algorithm. Hierarchical clustering of the patients was performed using Pearson correlation coefficient to define dissimilarity between patient expression profiles using only the probes associated with the genes included in the signature to be tested, obtaining two clusters of patients in each case. Kaplan-Meier curves were constructed for the two clusters of patients and the statistical significance of differences in survival probability between the two clusters was computed with the log-rank test. Univariate Cox analysis was performed to determine significant correlations between the expression profile of each individual gene represented on the chips and survival time.

### Quantitative real-time RT-PCR validation of microarray results

cDNA was obtained using random hexamers (Invitrogen, USA), dNTPs (Clontech, USA) and the reverse transcriptase Superscript II (Invitrogen) starting from totRNA extracted from microdissected material. Real-time PCR amplification was performed using a Syber green mix or a TaqMan primers and probes mix when available, in an ABI Prism 7700 instrument (Applied Biosystems, Foster City, CA, USA). Relative quantitation of target, normalized with an endogenous control 18s rRNA (Hs99999901_s1) was done using a comparative (Ct) method according to the manufacturer's instructions. For EGFR (Hs00193306_m1), TaqMan probes (Applied Biosystems) were used. ProbeFinder software (www.roche-applied-science.com) was used to design primers for the Syber green method. The sequences of the forward (Fw) and reverse (Rev) primers were: INHBA Fw (ctcggagatcatcacgtttg), Rev (ccttggaaatctcgaagtgc); RUNX1 Fw (tgcctccctgaaccactc), Rev (gatggttggatctgccttgta); TGFBR3 Fw (gatttcatcttcggcttgaaa), Rev (gctcaggaggaatagtgtgga); NOVA1 Fw (gggttcccatagacctggac), Rev (gaaaatactggccgtcttcg); ENPP2 Fw (tgatggcttacatgacacagaa), Rev (agtgagttggaacaggaatgg); POSTN Fw (gaaccaaaaattaaagtgattgaagg), Rev (tgacttttgttagtgtgggtcct); ESR1 Fw (ttactgaccaacctggcaga), Rev (atcatggagggtcaaatcca); NKX3-1 Fw (ctcagtccctactgagtactctttctc), Rev (cagtgaaatgtgtaacccttgc); HOXB13 Fw (aacccaccaggtcccttt), Rev (tgtacggaatgcgtttcttg); SFRP1 Fw (gctggagcacgagaccat), Rev (tggcagttcttgttgagcag); ERG Fw (gccaggtgaatggctcaa), Rev (agttcatcccaacggtgtct); NELL2 Fw (aagaactgcacatgcctgaa), Rev (tcaggatttgggcagattaga); BMP5 Fw (gcaataaatccagctctcatca), Rev (tgtttttgctcacttgtgttataatct); HOXD13 Fw (ggaacagccaggtgtactgc), Rev (cggctgatttagagccaca).

### Immunohistochemistry

Paraffin-embedded tissue sections were deparaffinized and hydrated according to standard procedures. Sections were subjected to antigen retrieval by boiling in EDTA (1 mM, pH 7.5) for 10 min, cooled, washed, and blocked in normal serum (from the same species from which the secondary antibody was produced). Frozen tissue sections were acetone-fixed and rehydrated prior to immunostaining and blocked in normal serum. The sections were then incubated with the primary antibody (for 1 hour at room temperature), followed by the incubation with the horse radish peroxidase (HRP)-conjugated secondary antibody for additional 30 minutes at room temperature. Diaminobenzidine (DAB) was used as a chromogene resulting in brown staining of positive cells. The nuclei were counterstained in blue using Harris hematoxylin. The antibodies were purchased as follows: NCL-C11 anti-multi-cytokeratin (Novocastra, UK), Keratin-903 anti-cytokeratin (cat. M 0630, Dako, USA), anti-human vimentin (cat. M 0725, Dako, USA), anti-periostin (cat. ab14041, Abcam, UK). For routine histopathological examination, 4-µm-thick frozen tissue sections were H&E stained according to standard procedures.

## Supporting Information

Figure S1 **Identification of tumor and stromal compartments.** Representative images of A, breast carcinoma and B, prostate carcinoma sections stained with multi-cytokeratin antibody, with tumor cells appearing in brown. C, representative image of breast carcinoma with the stromal compartment identified by brown staining using anti-vimentin antibody. Magnification: 400×.
(TIF)

Figure S2 **Laser capture microdissection.** Examples of stroma microdissection using LCM from A, normal breast tissue, B, breast carcinoma, C, normal prostate tissue and D, prostate carcinoma. Arrows indicate the epithelial compartment whereas arrowheads point to the stroma. Staining: H&E, magnification: 200×.
(TIF)

Figure S3 **Validation of gene expression.** qReal-time RT-PCR validation of genes identified by microarray analysis. A–B, breast cancer stromal genes, C–E, prostate cancer stromal genes. The strong induction of ESR1 is represented on a separate panel for graphical reason.
(TIF)

Table S1 **Histopathological classification of A, infiltrating breast ductal carcinoma and B, invasive prostate carcinoma patients used in the present study.**
(DOC)

Table S2 **Complete list of differentially expressed breast genes between tumor and normal stroma (FDR = 0.05).**
(XLS)

Table S3 **Complete list of differentially expressed prostate genes between tumor and normal stroma (FDR = 0.10).**
(XLS)
