# Supplementary figures and images for: Identification of Prognostic Molecular Features in the Reactive Stroma of Human Breast and Prostate Cancer
Source: PLoS One. 2011 May 18;6(5):e18640. doi: 10.1371/journal.pone.0018640 (PMC3097176; doi:10.1371/journal.pone.0018640)

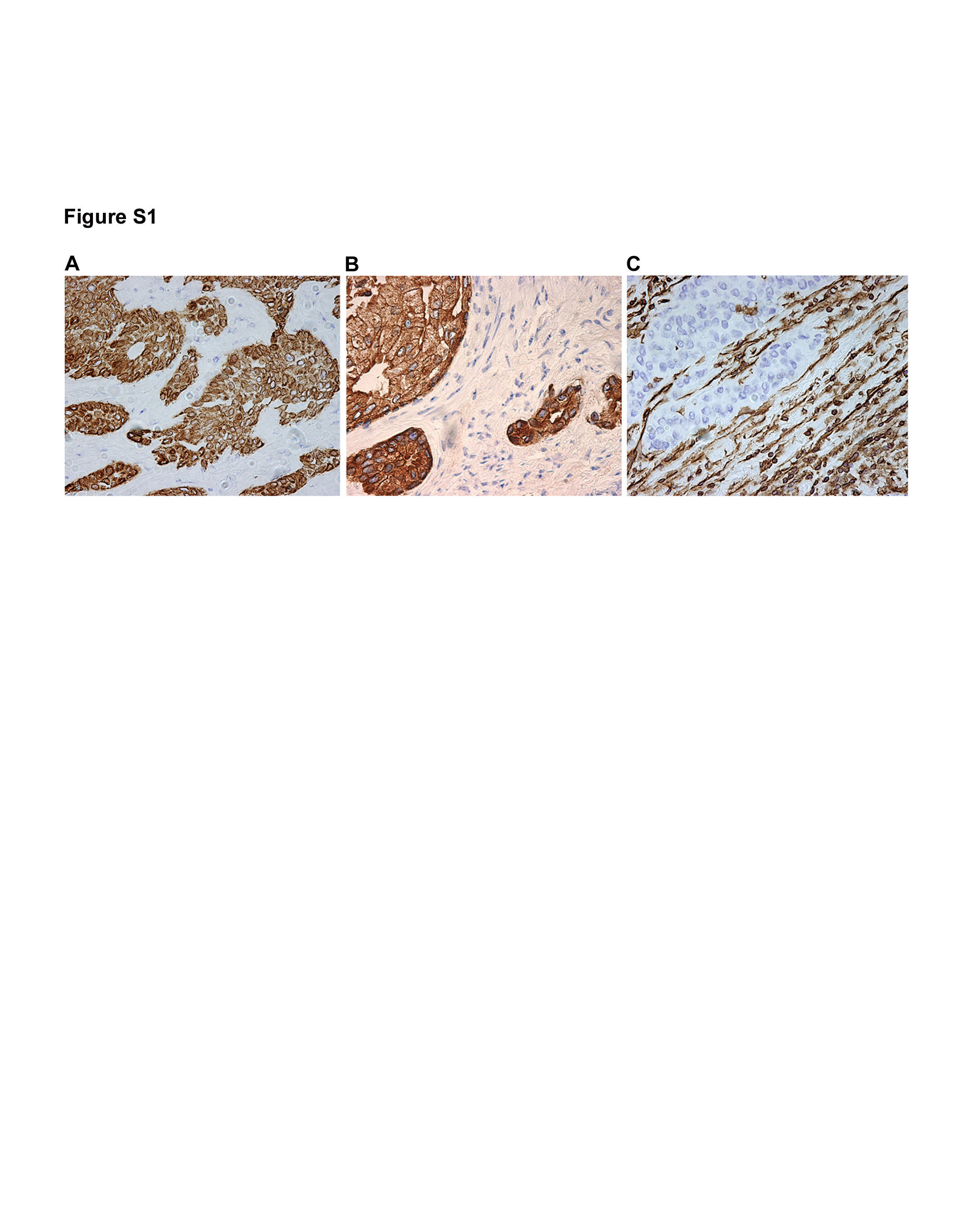

Supplement: Figure S1 — Identification of tumor and stromal compartments. Representative images of A, breast carcinoma and B, prostate carcinoma sections stained with multi-cytokeratin antibody, with tumor cells appearing in brown. C, representative image of breast carcinoma with the stromal compartment identified by brown staining using anti-vimentin antibody. Magnification: 400×. (TIF) [file pone.0018640.s001.tif]

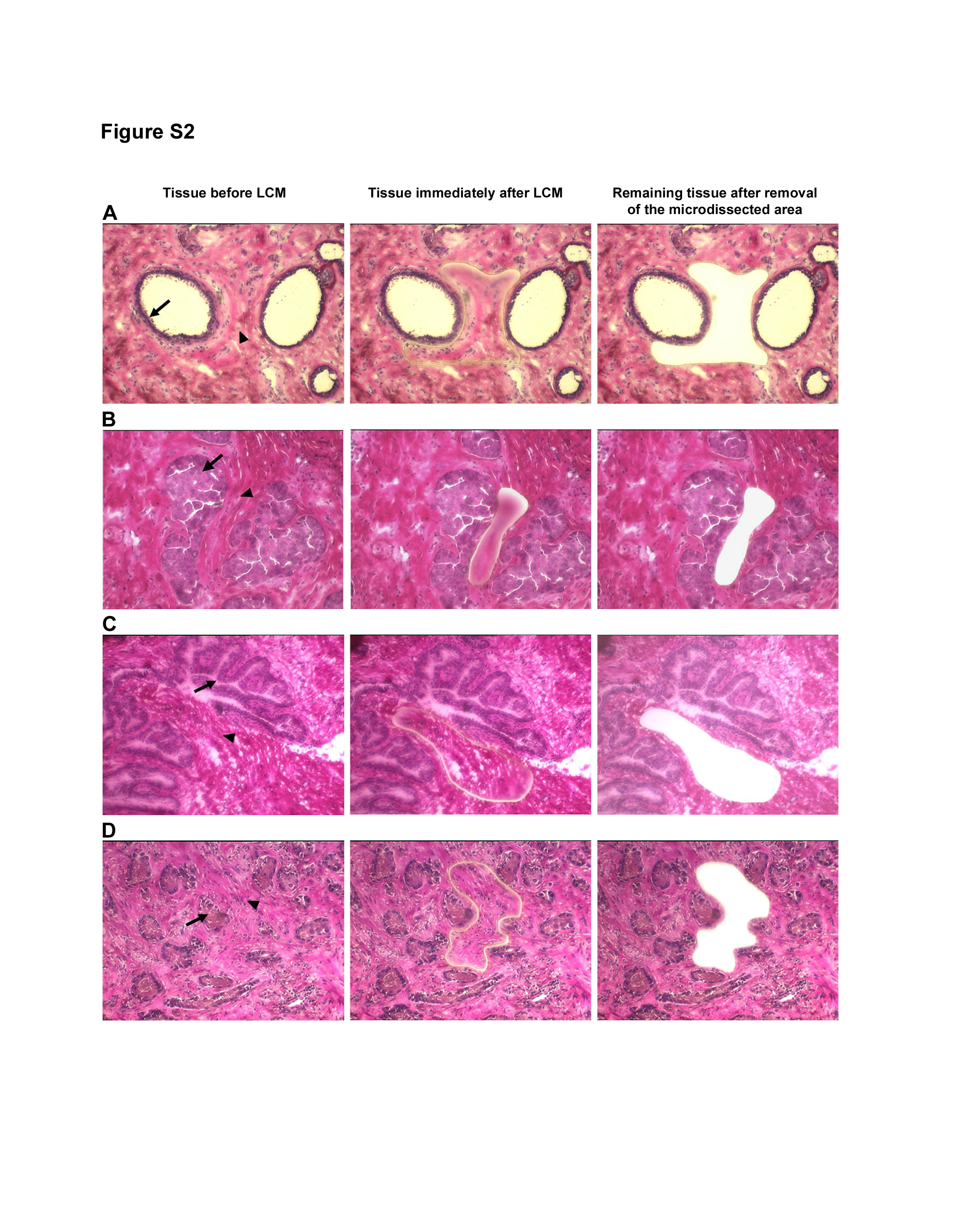

Supplement: Figure S2 — Laser capture microdissection. Examples of stroma microdissection using LCM from A, normal breast tissue, B, breast carcinoma, C, normal prostate tissue and D, prostate carcinoma. Arrows indicate the epithelial compartment whereas arrowheads point to the stroma. Staining: H&E, magnification: 200×. (TIF) [file pone.0018640.s002.tif]

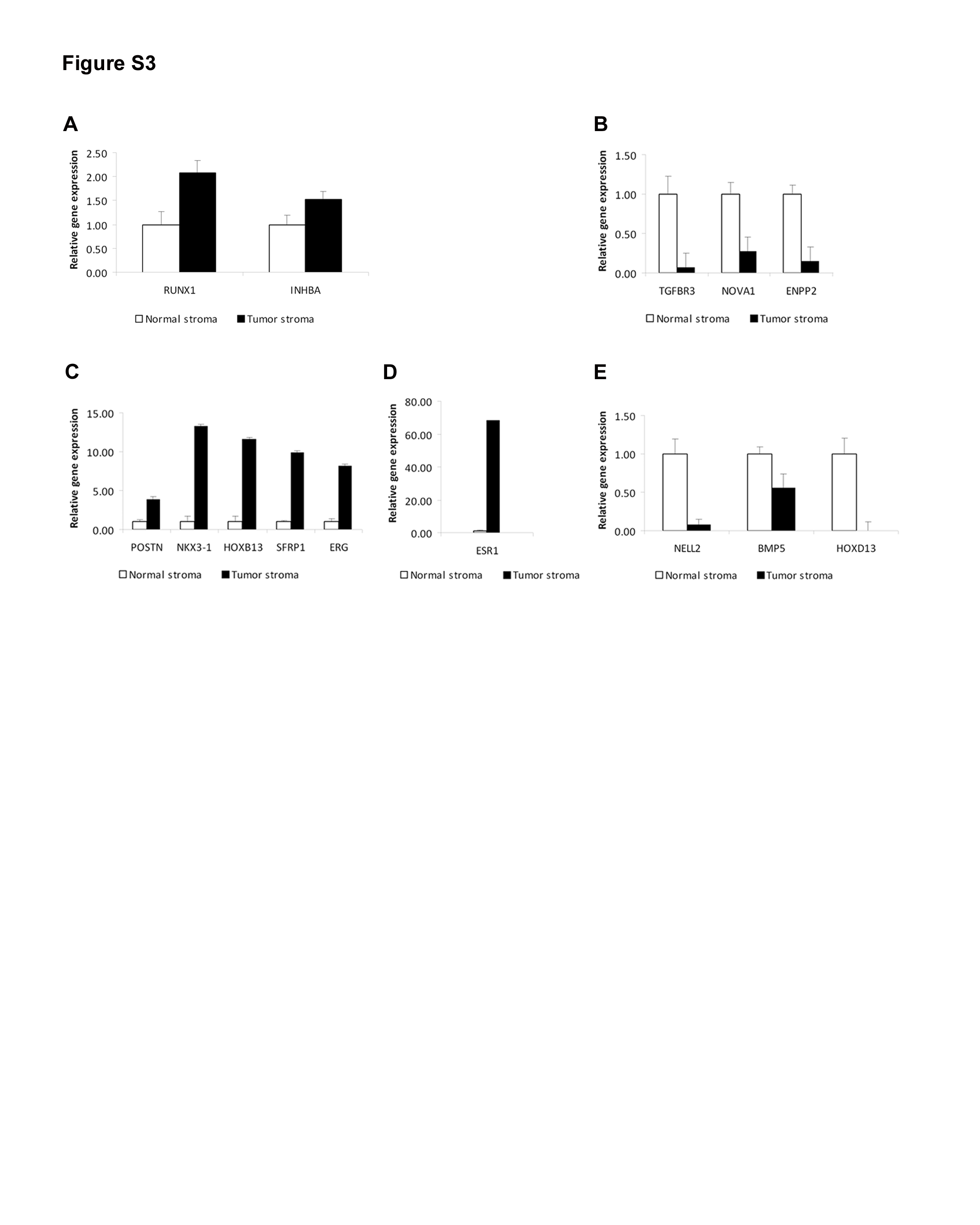

Supplement: Figure S3 — Validation of gene expression. qReal-time RT-PCR validation of genes identified by microarray analysis. A–B, breast cancer stromal genes, C–E, prostate cancer stromal genes. The strong induction of ESR1 is represented on a separate panel for graphical reason. (TIF) [file pone.0018640.s003.tif]
