# Supplementary material for: Identification of Prognostic Molecular Features in the Reactive Stroma of Human Breast and Prostate Cancer
Source: PLoS One. 2011 May 18;6(5):e18640. doi: 10.1371/journal.pone.0018640 (PMC3097176; doi:10.1371/journal.pone.0018640)
Supplement: Table S1 — Histopathological classification of A, infiltrating breast ductal carcinoma and B, invasive prostate carcinoma patients used in the present study. (DOC) [file pone.0018640.s004.doc]

**Table S1.** Histopathological classification of **A**, infiltrating breast ductal carcinoma and **B**, invasive prostate carcinoma patients used in the present study.

A

| **Tumor stage** | **Patient age** | **ER** | **PR** | **HER-2** |
| --- | --- | --- | --- | --- |
| pT1b, pN1 mi (sn), Mx | 51 | 100% | 100% | 0 |
| pT1c, pN2, Mx, R0, G2 | 56 | 90% | 95% | 0 |
| pT1c, pN1a (1/9), Mx, G2, R0 | 70 | 90% | 70% | 1+ |
| pT3, pN1a (1/9), Mx, G2, R0 | 90 | 100% | 0% | 2+ |
| pT2, pN1a (3/14), Mx | 57 | 0% | 0% | 0 |
| pT2, pN0, pMx | 47 | 100% | 100% | 0 |

B

| **Tumor stage** | **Patient age** | **Gleason** |
| --- | --- | --- |
| pT2c, pN0 | 56 | 7 |
| pT3a, pN0, Mx | 58 | 4+4=8 |
| pT2c, pN0 (0/7), Mx, R1 | 60 | 7 |
| pT3a, pN0 (0/6), Mx | 74 | 7 |
| pT2c, pN0 (0/8), Mx, R1 | 62 | 7 |
| pT3a, pN0, Mx | 75 | 4+4=8 |
